# Radial nerve injury associated with humeral shaft fracture: a retrospective study

**DOI:** 10.1590/1413-78522015230100823

**Published:** 2015

**Authors:** Flávia Pessoni Faleiros Macêdo Ricci, Rafael Inácio Barbosa, Valéria Meirelles Carril Elui, Cláudio Henrique Barbieri, Nilton Mazzer, Marisa de Cássia Registro Fonseca

**Affiliations:** Universidade de São Paulo, Faculdade de Medicina de Ribeirão Preto, Ribeirão Preto, SP, Brasil, Faculdade de Medicina de Ribeirão Preto da Universidade de São Paulo, Ribeirão Preto, SP, Brasil

**Keywords:** Humeral fractures, Radial nerve, Epidemiology, descriptive

## Abstract

**Objective:**

To determine the profile of patients with humeral diaphyseal fractures in a tertiary hospital.

**Methods:**

We conducted a survey from January 2010 to July 2012, including data from patients classified under humeral diaphyseal fracture (S42.3) according to the International Classification of Diseases (ICD-10). The variables analyzed were: age, gender, presence of radial nerve injury, causal agent and the type of treatment carried out.

**Results:**

The main causes of trauma were car accidents. The radial nerve lesion was present in some cases and was caused by the same trauma that caused the fracture or iatrogenic injury. Most of these fractures occurred in the middle third of humeral diaphysis and was treated conservatively.

**Conclusion:**

The profile of patients with fracture of humeral shaft, in this specific sample, was composed mainly of adult men involved in traffic accidents; the associated radial nerve lesion was present in most of these fractures and its cause was strongly related to the trauma mechanism. Level of Evidence II, Retrospective Study.

## INTRODUCTION

Traumatic injuries of the upper limbs can cause damage, often permanent, affecting both the functional day-to-day activities and former professional activities.^1^^,^ 2 The vast majority of injuries of peripheral nerves affect the upper limbs and the mostly affected nerve is the radial nerve, the most common cause being diaphyseal fractures of the humerus.^3^^-^ 5 The humeral diaphyseal fractures account for approximately 3% of all orthopedic injuries.^6^^-^ 9 Its relationship with radial nerve injury is due to anatomical factors, because this nerve is fixed and near the bone in the middle third transition to the distal third of the humerus.^7^^,^ 10 ^,^ 11

The classic biomechanical consequence of the radial nerve paralysis is the inability to extend the wrist, loss of extension of the fingers in the metacarpophalangeal joints and inability to extend and abduct the thumb. Also known as "wrist drop", the deformity that is established after a radial nerve injury represents a significant functional damage to the hand, since the inability to extend and stabilize the wrist prevents proper use of extrinsic flexors for hand closing, thus, weakening and lowering the hold and diminishing coordination.^12^^,^ 13

Some recently published retrospective studies show incidence of radial nerve injury associated with diaphyseal fracture of the humerus between 11% and 18%, indicating car accidents as the leading cause of diaphyseal fracture of the humerus,^4^^,^ 6 ^,^ 9 ^,^ 14 ^-^ 16 highlighting as main affected population young men and older women.^3^^,^ 7 ^,^ 9

This study aimed to determine the profile of patients with diaphyseal fracture of the humerus treated in a regional referral tertiary health care hospital of the Unified Health System (SUS) located in a Brazilian city with over 600,000 inhabitants and a regional population of approximately 305,000 inhabitants.

## MATERIALS AND METHODS

This was a descriptive retrospective study, aimed at collecting data on diaphyseal fracture of the humerus between January 2010 and July 2012, at the *Hospital das Clínicas da Faculdade de Medicina de Ribeirão Preto da Universidade de São Paulo*, Ribeirão Preto, SP, Brazil. We included data of patients classified under diaphyseal fracture of the humerus (S42.3) according to the International Classification of Diseases (ICD-10). The variables analyzed were age, gender, presence of radial nerve injury, causal agent and the type of treatment performed.

## RESULTS

**Figure 1.**
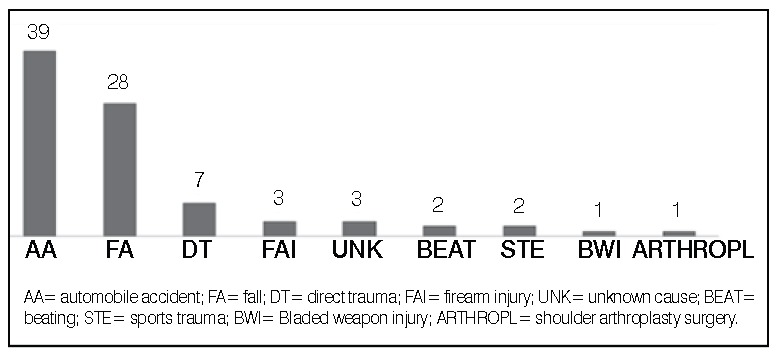
Causes of trauma.

During the period covered by the study 117 patients were found in total. Of these, 31 were excluded initially, 14 to be related with other types of fracture and 17 of serious polytraumatized patients who died before a proper assessment to verify the presence or absence of radial nerve injury. Thus, the sample used in this study was composed by 86 patients. Demographic data of the patients are described in Table 1. The main causes of trauma were motor vehicle accidents and falls. (Figure 1)

**Table 1.** Mean age and gender distribution.

	N	Mean age (years old)	%
Total	86	35	100
Men	65	32.4	75.6
Women	21	39.5	24.4

**Figure 2.**
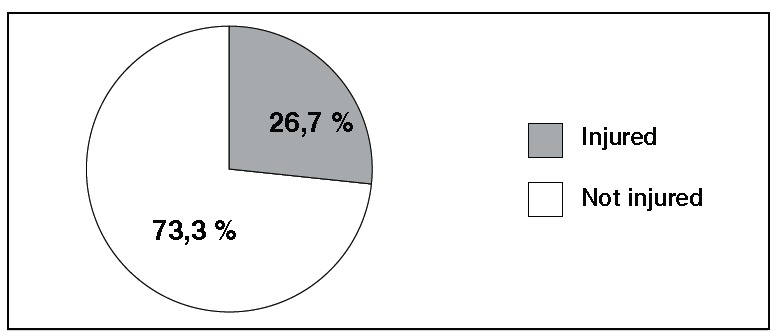
Incidence of radial nerve injury among cases of diaphyseal fracture of the humerus.

**Figure 3.**
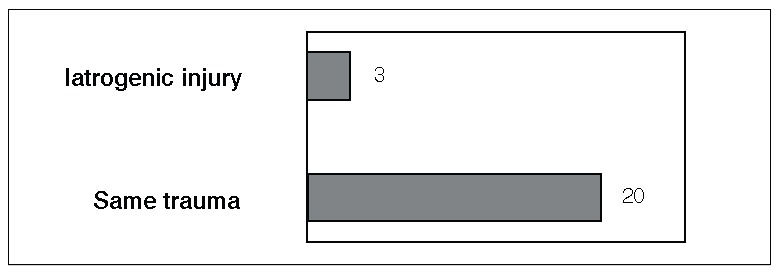
Causes of injury of the radial nerve associated to diaphyseal fracture of the humerus.

**Figure 4.**
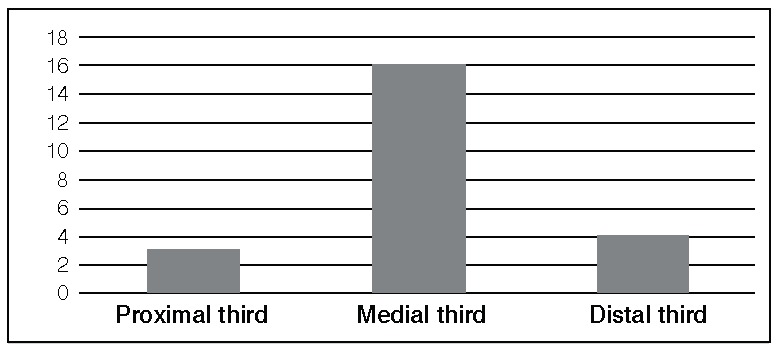
Location of diaphyseal fracture of the humerus among cases with radial nerve injury.

**Figure 5.**
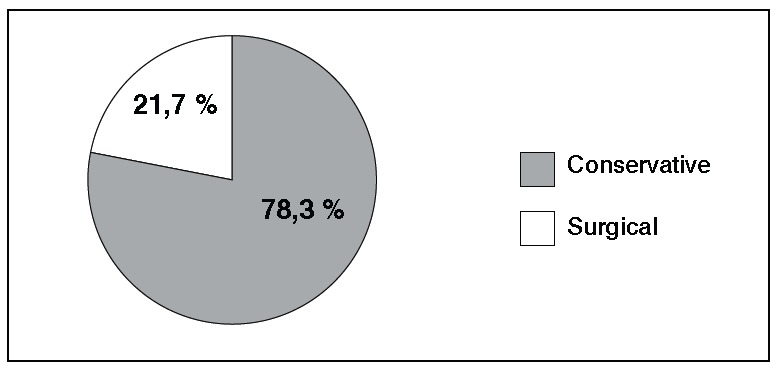
Type of treatment chosen for radial nerve injuries associated with diaphyseal fracture of the humerus.

The radial nerve injury was present in cases of diaphyseal fracture of the humerus (Figure 2) and had been caused by the same trauma that caused the fracture or iatrogenic injuries. (Figure 3) Most fractures associated with radial nerve injury happened in the middle third of the humeral shaft. (Figure 4) One patient had a brachial plexus injury associated with ipsilateral radial nerve injury. Most of these injuries were treated conservatively. (Figure 5) When the option was surgery, the procedures performed were graft, neurotization or neurolysis. One of the cases of radial nerve injury progressed to limb amputation due to vascular complications.

## DISCUSSÃO

The radial nerve damage can lead to severe disability due to the key role that this nerve has on proper positioning of the wrist during the functional use of the hand.^12^^,^ 13 Several retrospective and epidemiological studies^4^^,^ 6 ^,^ 9 ^,^ 14 ^-^ 16 showed a strong association between injury of the radial nerve and the diaphyseal fracture of the humerus.

In this study, diaphyseal fracture of the humerus had as main cause automobile accidents, accounting for 45.3% of cases, mainly involving men (75.6%) with a mean age of 32.4 years old. The incidence of associated radial nerve injury was 26.7% and showed to be slightly higher than the values previously described in the literature (11% to 18%).

Our results corroborate those reported by Tsai *et al.*,^17^ who conducted a similar study in Taiwan. The authors reviewed the demographic variables, incidence of radial nerve injuries and cause of trauma, among others, and also found as most affected population young men, with mean age 31.7 years old. Moreover, as in our study, the authors found as the main cause of trauma automobile accidents (63.2%), followed by some sort of fall (36.4%).^17^

On the other hand, in a Sweden population, an epidemiological study on diaphyseal fracture of the humerus had totally opposite results than ours.^18^ Ekholm *et al.*18 found as most affected population older women (61%), with a mean age of 68.2 years old. The main cause of trauma was falls, accounting for 76% of cases, and car accidents accounting for only 5% of trauma. These findings reflect the low incidence of high-energy trauma in Sweden, the opposite of what happens in Brazil.

In an epidemiological study on traumatic peripheral nerve damage in general, Ciaramitaro *et al.* 3 found that over 80% of these injuries occur in the upper limbs and that the radial nerve was the most affected. Of all the injuries, 15% were iatrogenic, and all these occurred during orthopedic surgery and the strongest association was to surgery to humeral osteosynthesis. Among the injuries of the radial nerve, and 16% were due to iatrogenic causes. This value is similar to what was found in the present study in which three of the 23 radial nerve injuries were iatrogenic, which corresponds to 13%. These data suggest that the vast majority of the radial nerve injury is associated with the mechanism of the trauma itself.

Regarding the treatment of these injuries we observed that the vast majority was treated conservatively. Perhaps this finding is due to the fact that in this study the majority of fractures was closed and the analysis of variables was performed only once and there was no monitoring of the progress of cases. Recent literature suggests that when there are clinical signs of radial nerve injury after closed fractures of the humeral diaphysis the best approach to be taken initially is the observation and reassessments to determine new approaches.^6^^,^ 9 ^,^ 14 ^,^ 16

In Brazil, violence is responsible for most of the morbidity and mortality, and the second leading causes of deaths from external causes are traffic accidents. Although there is a declining trend in the number of injuries and traffic-related deaths in recent years, this is a major public health problem in Brazil, due to its has high personal and social cost.^19^ In an attempt to minimize this problem several measures have been taken by the Brazilian government. Besides the Brazilian Traffic Code, which provides penalties for violations for car racing, drunk driving or not using the seat belt, other projects have been developed and new laws approved.^19^ In 2002, the Ministry of Health approved the Project Reducing the Morbidity and Mortality of Traffic Accidents - Mobilizing Society and Promoting Health (*Projeto de Redução da Morbimortalidade por Acidentes de Trânsito - Mobilizando a Sociedade e Promovendo a Saúde*) in order to implement, in selected urban areas, health promotion and accident prevention actions. The city where we developed the present study was among the chosen cities for the project development.^20^ More recently, in September 2010, the National Committee for Health, Safety and Peace in Traffic Mobilization (Comitê Nacional de Mobilização pela Saúde, Segurança e Paz no Trânsito) proposed the *National Plan for Accident Reduction and Road Safety for the 2011-2020 decade (Plano Nacional de Redução de Acidentes e Segurança Viária para a década 2011-2020)*, which determined actions based on surveillance, education, health, infrastructure and vehicular safety.^21^ Regarding new laws in July 6, 2011 Law 12,436, which prohibits practices that encourage increased speed for professional motorcyclists was enacted.^22^ On August 9, 2012, the Secretariat of Health Surveillance published Ordinance N 22 in order to establish guidelines for the transfer of financial resources for the implementation and strengthening of National Policy of Health Promotion (*Política Nacional de Promoção da Saúde*), in order to foster surveillance actions and prevention of violence and accidents, prevention of injuries and deaths in traffic, and promoting peace in traffic.^23^

## CONCLUSION

The profile of patients with diaphyseal fracture of the humerus, in this particular sample, was composed mainly of adult men involved in traffic accidents. The associated radial nerve injury was present in most of these fractures and their cause was strongly related to trauma mechanism. Traffic accidents are preventable and should continue to be emphasized in public health policies. Moreover, these data can help in preparing the patient treatment plan with this particular injury, since it predominantly affects young individuals at working age who need to return to work as soon as posible.
